# CON: Serum biomarker monitoring should not replace primary antifungal chemoprophylaxis in patients with acute leukaemia receiving systemic anti-cancer therapy

**DOI:** 10.1093/jacamr/dlac081

**Published:** 2022-07-22

**Authors:** Alex Howard, William Hope

**Affiliations:** Liverpool University Hospitals NHS Foundation Trust, Prescot Street, Liverpool, L7 8XP, UK; Antimicrobial Pharmacodynamics and Therapeutics, University of Liverpool, Liverpool, L69 3BX, UK

## Abstract

Primary antifungal chemoprophylaxis (PAC) is the widespread strategy of choice for the prevention of invasive fungal disease in patients with acute leukaemia (AL). Twice-weekly monitoring of the serum biomarkers (SBM) galactomannan and 1,3-β-d-glucan has been proposed as an alternative prevention strategy to PAC for these patients. This paper outlines the arguments for why PAC should remain as the standard of care in AL, instead of switching to twice-weekly SBM. Arguments put forward in favour of PAC are the strength of evidence for its safety, cost-effectiveness and adaptability, and its adoption by multiple international guidelines as standard of care. The potential implications of PAC for drug interactions and antifungal resistance are also discussed. The drawbacks of twice-weekly SBM are appraised, including missed or delayed diagnoses, unnecessary investigations, deferral of systemic anti-cancer therapy and increased pressure on laboratory services.

## Introduction

Individuals undergoing systemic anti-cancer therapy (SACT) for acute leukaemia (AL) are at high risk of developing invasive fungal disease (IFD), a group of infections associated with significant morbidity and mortality.^1^ Primary antifungal chemoprophylaxis (PAC) in AL has therefore become widespread practice that is recommended by European Conference on Infections in Leukaemia (ECIL) and Infectious Diseases Society of America (IDSA) guidelines.^2,3^ However, increased uptake of the serum fungal biomarkers galactomannan (GM) and 1,3-β-d-glucan (BDG) in the diagnosis of IFD has provoked interest in the potential of these tests as alternatives to PAC. This paper argues that twice-weekly serum biomarker monitoring (SBM) with GM and BDG should not replace PAC as the primary IFD prevention strategy in AL.

## The status quo

The evidence to date well illustrates the spectrum of threats that IFD presents to patients with haematological malignancy and HSCT, and the efficacy of PAC in combating them. When deployed in randomized controlled trials, fluconazole PAC significantly reduced the incidence of IFD and associated mortality in neutropenic cancer patients and bone marrow transplant recipients.^4,5^ Mould-active PAC was subsequently shown to be even more efficacious than fluconazole for this indication, demonstrating the importance of a PAC agent with activity against both *Candida* species and moulds in vulnerable patients.^6,7^ The diagnostically challenging nature of IFD means that it cannot be known with certainty whether these findings apply to all centres, as the incidence of IFD in AL may vary geographically, and true IFD rates are difficult to establish without widespread use of post-mortem diagnosis. Given the severe consequences of IFD, however, current evidence suggests that PAC should be regarded as the preferable option unless reliable, well-powered local data prove otherwise.

Posaconazole is now, therefore, the most widely used PAC agent in AL and HSCT. The agent is well absorbed and well tolerated following oral administration and serious adverse events are rare.^8–10^ Therapeutic drug monitoring (TDM) is also available in most developed healthcare settings either locally or via a regional reference laboratory to help optimize effectiveness and prevent concentration-dependent therapeutic failure. Economic analyses have revealed posaconazole PAC to be cost-effective, an effect driven by avoidance of treatment costs and increased length of hospital stays associated with IFD.^11^ Potentially severe drug interactions can prevent concomitant use of posaconazole with some SACT agents such as vincristine and dasatinib, but echinocandins, polyenes and isavuconazole are alternative PAC options with evidence for their efficacy.^12–15^ An increased global focus on antifungal stewardship means that the question of whether PAC could increase the prevalence of antifungal-resistant organisms is relevant. Importantly for antifungal stewardship programmes, however, the limited evidence that does exist for increasing triazole resistance is both conflicting and prone to reporting bias.^16–18^ When breakthrough IFD on prophylaxis is suspected, diagnosis is particularly challenging because the presence of PAC is likely to reduce the sensitivity of serum fungal biomarkers.^19^ Given the extensive experience of most centres in using PAC, however, diagnostic algorithms often account for this by using pre-emptive, fever-driven treatment approaches.

## The proposed alternative

In patients with cancer, the accuracy of serum BDG and GM for diagnosis of IFD have varied in meta-analyses owing to the significant heterogeneity of included studies. The best sensitivity and specificity outcomes for serum BDG in meta-analysis of IFD diagnosis have been 80% and 63%, respectively.^20^ Diagnostic accuracy of serum GM in immunocompromised patients varies with optical density index (ODI) cut-off; 0.5 ODI yielded 82% and 81% sensitivity and specificity, respectively; 1.0 yielded 72% and 88%; 1.5 yielded 61% and 93%.^21^ Deployment of the tests in parallel as twice-weekly SBM may improve overall performance, but combining them is under-studied in this cohort, and is still likely to result in missed diagnoses. This is as much due to test design as performance; serum GM does not detect *Candida* and neither test detects Mucorales^22^ Conversely, false positives are likely to result in unnecessary delays to crucial SACT,^23^ and unnecessary investigations exposing the patient to ionizing radiation through CT scanning or invasive procedures like bronchoscopy.

The utility of SBM is also likely to be limited by laboratory resource pressures, particularly given that a strategy of SBM is likely to increase the number of BDG and GM tests being requested. Many local microbiology laboratories rely on transporting samples to regional reference mycology laboratories for such tests, a process that incurs additional cost, prolonged turnaround time and inefficiency. Even where such tests are offered in house, laboratories are under increasing clinical pressure to deliver a range of tests that compete for staff time and resources. The overall outcome in both scenarios is increased turnaround times, potentially negating the benefit of early detection and missing the opportunity to implement timely antifungal treatment.^24^ Clinical pressures may also favour the use of PAC; as clinical pressures on healthcare systems continue to increase with population, early discharge of stable but vulnerable patients may be a necessity. In that scenario, clinical teams may be reluctant for patients to be discharged or managed at home without cover for environmental fungal pathogens that are more abundant in the community.^25^

There is an evidence base for the cost-effectiveness of BDG as an antifungal stewardship tool, with savings predominantly driven by minimizing antifungal use. This evidence, however, pertains to enabling earlier discontinuation of pre-emptive antifungal treatment rather than as an alternative to PAC. It is therefore unknown whether the pharmacy saving of reducing PAC prescribing would be sufficient to offset increased laboratory costs in addition to the investigation, management and prolonged hospitalization of patients with IFD managed reactively rather than preventatively.

## Conclusions

Preventing IFD using PAC has proven to be a safe, cost-effective and adaptable strategy in neutropenic patients with haematological malignancy and/or bone marrow transplant, with a strength of evidence reflected by its support in international guidelines. Replacing PAC with twice-weekly SBM in AL may expose patients to increased risk through missed or delayed diagnoses, unnecessary investigations and deferral of SACT, as well as putting increased pressure on laboratory services already experiencing unprecedented demand. The strategy is also yet to be proven to be cost-effective or to reduce the selection of antifungal resistance, and it is less practical for patients being managed in the community. Patients with AL should continue to be administered PAC to prevent IFD instead of twice-weekly serum GM and BDG monitoring.

## Transparency declarations

William Hope holds or has recently held research grants with UKRI, EU, F2G, Spero Therapeutics, Antabio, Pfizer, Bugworks, Phico Therapeutics, BioVersys, GARDP and NAEJA-RGM. He is (or has recently been) a consultant for Appili Therapeutics, F2G, Spero Therapeutics, NAEJA-RGM, Centauri, Pfizer, Phico Therapeutics, Pulmocide, Amplyx, Mundipharma Research Ltd and VenatoRx. He is a member of the Specialist Advisory Committee for GARDP and the Specialty National Co-lead for Infectious Diseases for the National Institute of Health Research (NIHR). All other authors: none to declare.
